# Successful treatment of a symptomatic L5/S1 discal cyst by percutaneous CT-guided aspiration

**DOI:** 10.4103/2152-7806.68338

**Published:** 2010-08-10

**Authors:** Hormuzdiyar H. Dasenbrock, Sudhir Kathuria, Timothy F. Witham, Ziya L. Gokaslan, Ali Bydon

**Affiliations:** 1 School of Medicine, Johns Hopkins University, 600 N Wolfe St., Baltimore, MD 21287; 2 Department of Radiology and Radiological Science, Johns Hopkins University, 600 N Wolfe St., Baltimore, MD 21287; 3 Department of Neurosurgery, Johns Hopkins University, 600 N Wolfe St., Baltimore, MD 21287

**Keywords:** CT-guided aspiration, disc cyst, discal cyst, intervertebral disc, percutaneous spinal interventions

## Abstract

**Background:**

Discal cysts are a rare cause of lumbar radiculopathy. Benefits of percutaneous computed tomography (CT)-guided aspiration of the cyst include decreased rate of infection, avoidance of general anesthesia, and quicker recovery. However, since the publication of a case of cyst recurrence after CT-guided aspiration, few have utilized this potentially valuable technique.

**Case Description:**

We present a patient with a discal cyst arising from the L5/S1 disc causing right S1 radiculopathy. He underwent percutaneous CT-guided aspiration with substantial improvement in his radicular pain with 19 months of follow-up. His improvement was measured quantitatively using the Japanese Orthopedic Association scale: 6/15 pre-procedure, 15/15 post-procedure.

**Conclusion:**

Percutaneous CT-guided aspiration of discal cysts may be a valid initial treatment option for this condition. Patients who do not respond or who have a recurrence can subsequently be treated by surgical excision.

## INTRODUCTION

Degenerative conditions of the lumbar spine are common causes of radiculopathy. A number of rare conditions exist that may present identically; discal cysts, intraspinal extradural cysts that communicate with the intervertebral disc, are a rare cause of radiculopathy. First reported in English in 1999,[13] less than 100 patients with discal cysts have been published until now, primarily in case reports and case series.[1] However, as discal cysts are a unique pathological entity, an understanding of the different therapeutic options for this condition is important.

Percutaneous aspiration of discal cysts has many advantages, including avoiding the risks and complications of open surgery, avoiding general anesthesia, and a faster recovery. A report of recurrence after percutaneous computed tomography (CT)-guided aspiration[9] has led some to recommend surgical excision as the preferred treatment option.[1] The true recurrence rate after CT-guided aspiration remains unknown due to the rarity of the procedure and the short follow-up period in the reports.[9,12,17] Recurrence has also been described after surgical excision.[14] We report a case of a symptomatic discal cyst that was aspirated percutaneously under CT guidance, with good pain control 19 months later.

## CASE

### Presentation and examination

A 37 year-old Caucasian male presented with a 7-month history of mild low-back pain and severe right S1 radiculopathy. He had failed conservative management with oral steroids and physical therapy. Physical examination was remarkable for a positive straight leg raise on the right side at 70°.

### Imaging

**Figure 1:**
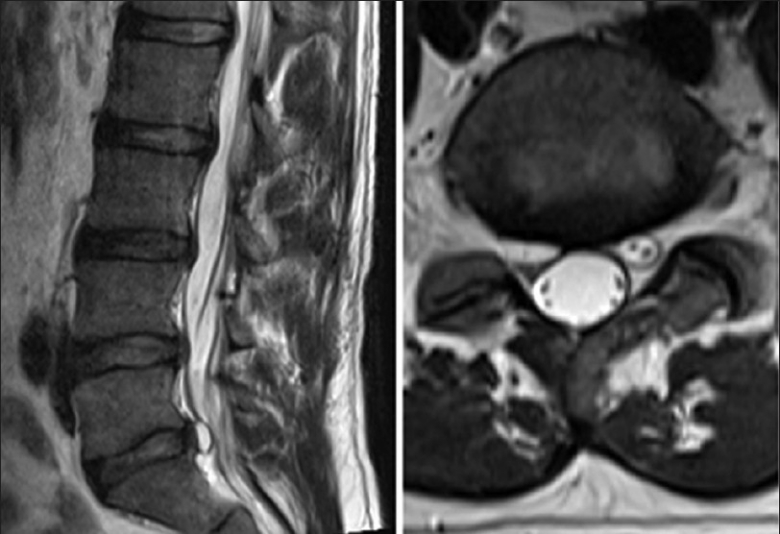
T2-weighted magnetic resonance image of a patient with right-sided S1 radiculopathy. Sagittal (left) and transaxial (right) views at the level of the L5-S1 disc demonstrate a T2 hyperintense cystic structure (arrow) extending into the right ventrolateral extradural space

**Figure 2:**
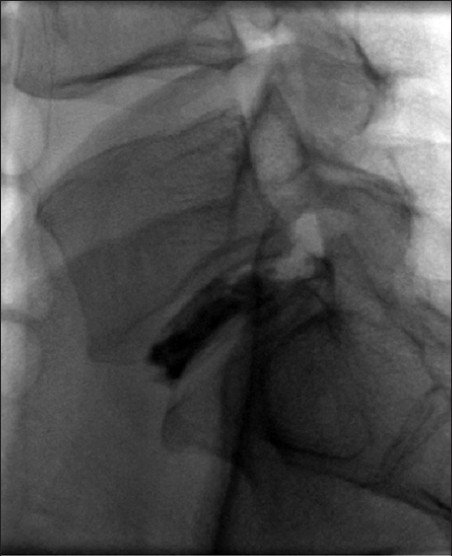
Fluoroscopy-guided L5-S1 discogram was performed from the contralateral (left) side. Lateral view reveals contrast filling into an intraspinal cyst-like outpouching from the disc

A magnetic resonance imaging (MRI) scan showed a cystic lesion at the L5-S1 level originating from the right lateral disc causing right lateral recess stenosis and posterior displacement of the traversing nerve root (S1). The cyst was hyperintense on T2-weighted and hypointense on T1-weighted images, with a rim of contrast enhancement present [Figure 1]. A contralateral L5-S1 discogram revealed a fissure in the disc through which contrast extruded from the disc to a cyst-like intraspinal extradural outpouching [Figure 2], consistent with a discal cyst.

### Intervention

**Figure 3:**
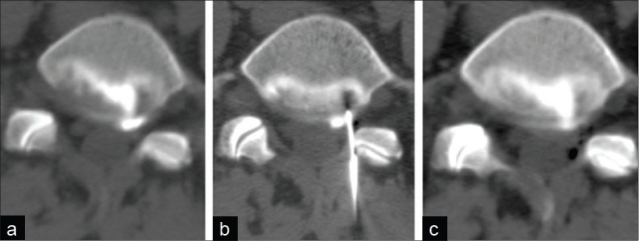
Transaxial computed tomography (CT) scan at the L5-S1 level. Before the CT-guided aspiration, the discal cyst is hyperdense (a). After CT-guided needle placement (b), the contents of the cyst were aspirated. Subsequently, the cyst is substantially smaller (c)

The discal cyst was treated with CT-guided aspiration. An 18-gauge needle was advanced into the center of the cyst. Transaxial CT images confirmed the appropriate placement of the needle, and a small amount of fluid was aspirated. Subsequent CT imaging showed a significantly smaller cyst [Figure 3].

### Outcome

The patient reported significant relief of his radiculopathy with excellent pain control documented 19 months post-procedure. His clinical outcome was quantitatively measured using the Japanese Orthopedic Association scale: his preoperative score was 6/15, and his postoperative score improved to 15/15.

## DISCUSSION

Discal cysts occur most commonly in young Asian men.[1] Whether this represents a differential susceptibility to develop discal cysts or an unidentified risk factor is not known. The fact that discal cysts are seen at slightly rostral levels than disc herniation, can present in the absence of additional spinal pathology and most commonly affect young males, may suggest a pathophysiology distinct from that of degenerative conditions.[1]

There are several theories regarding the etiology of discal cysts. Some have suggested that either disc herniation or trauma leads to the formation of an epidural hematoma, and deficits in its re-absorption lead to the cyst formation.[2] Others have hypothesized that focal degeneration of the intervertebral disc leads to leakage of fluid, which elicits a marked inflammatory reaction, causing the formation of a pseudomembrane which forms the wall of the discal cyst.[8] Finally, Marshman *et al*. have argued that discal cysts are synonymous with posterior longitudinal ligament/ annulus fibrosus ganglion cysts, likely developmental or degenerative in origin.[15]

Discal cysts have no distinguishing features on history or examination; the diagnosis is best made with MRI, which typically reveals a cystic sac that is hypointense on T1-weighted and hyperintense on T2-weighted images, with a rim of contrast enhancement after gadolinium administration. However, cysts with hemorrhage may be hyperintense on both T1- and T2-weighted images, and the rim of contrast enhancement is not always present.[14] Discography is used diagnostically to document a connection between the intervertebral disc and the cyst.

The optimal treatment of discal cysts remains debated. The natural history of this condition has yet to be defined, providing little guidance on prognosis. Spontaneous regression of a discal cyst has been reported.[5] The successful resolution of discal cysts has been described with both epidural and fluoroscopy-guided intra-discal steroid injections.[3,6] Although most reported cases have failed conservative therapy, it is not clear if this solely represents a publication bias or if discal cysts are less responsive to conservative therapy. However, an initial trial of conservative therapy should be pursued in patients without neurologic deficits.

Percutaneous treatment of discal cysts by CT-guided aspiration has many potential advantages, including decreased infection rates, avoidance of general anesthesia, and faster recovery. Surgical excision can be performed on those who fail initial percutaneous drainage procedures.

Koga *et al*. first reported the successful treatment of a discal cyst by CT-guided puncture and steroid injection.[12] Subsequently, Norman *et al*. also reported the usage of this approach,[17] and a larger series with 8 patients was later published by Kang *et al*.[9] Nine of the 10 patients in these reports reported an excellent clinical outcome, with no residual post-procedural symptoms. Moreover, no complications from the aspiration have been described. However, 1 patient in Kang *et al*.’s series had a recurrence 3 months postoperatively, which required subsequent surgical removal. Since the publication of this recurrence of the discal cyst after aspiration, there have been no further reports of the usage of this approach, and some have argued that surgical excision should be the treatment of choice for this condition.[1] Although most patients with discal cysts treated surgically, either open[2,4,8,11,13–16] or endoscopic,[7,10] have reported good outcomes, recurrence has also been reported after surgical excision.[14]

The reports of CT-guided percutaneous aspiration published to date are limited by a small sample size and relatively short mean follow-up period, ranging from 6 to 14.7 months.[9,12] Thus, it is difficult to estimate the true recurrence rate of discal cysts after CT-guided aspiration. Although surgical excision is the most common therapeutic modality used for symptomatic discal cysts, CT-guided percutaneous aspiration is another valid initial therapeutic option.
